# Medication Adherence Among Post-stroke Elderly Patients: A Cross-Sectional Study

**DOI:** 10.7759/cureus.83052

**Published:** 2025-04-26

**Authors:** Madhuri Mahavadia, Apurva Agrawal

**Affiliations:** 1 Department of Pharmacology, Rabindra Nath Tagore Medical College and Hospitals, Udaipur, IND

**Keywords:** adherence, arms, elderly, medication, stroke

## Abstract

Introduction

The rising burden of noncommunicable diseases is a significant health-related issue, especially in developing economies. Stroke is one of the major causes of mortality and morbidity in the geriatric population. Prevention of disability and mortality in post-stroke patients requires consistent medication adherence. Poor medication adherence is associated with poor outcomes, increased costs, and life-threatening complications.

Aim

The purpose of this study is to determine the level of medication adherence among post-stroke elderly patients and explore the relationship between medication adherence and various demographic, medication, and ailment-related factors.

Methods

A cross-sectional observational study was conducted in the geriatric outpatient department of a tertiary care hospital in Rajasthan, India. Post-stroke patients aged 60 years or older were included. Patients were interviewed, and data were entered into a structured and pre-validated questionnaire. Questions included demographic, medication, and ailment-related information, and the Adherence to Refills and Medications Scale (ARMS) scale was used. An ARMS score of ≤ 13 was considered adherent.

Results

A total of 100 elderly post-stroke patients were enrolled. The mean age of patients was 67.58 ± 6.78 years. Polypharmacy was present in 81% of patients, and 33% were found to be adherent to their medications (ARMS ≤ 13). The mean ARMS score was 15.72. Family type (p=0.0309), level of education (p=0.0016), and type of occupation (p=0.0323) significantly affected medication adherence. Person responsible for drug administration (p=0.0020) and patient’s positive beliefs (p=0.0398) also significantly affected adherence.

Conclusion

Medication adherence among post-stroke elderly patients in India is significantly low. Lack of education, unemployment, reduced familial support, patients' dependence on family members, and misconceptions about medications affect adherence. Physicians must remain vigilant and emphasize addressing patients’ fears and false beliefs about medications.

## Introduction

In this modern era of rapid advancements in medical science, where technology is progressing at a remarkable pace, challenges in the field of medical science remain significant. As the world continues to make progress, it also faces the increasing burden of non-communicable diseases, particularly chronic diseases [1]. In 2021, they accounted for at least 43 million deaths worldwide, representing 75% of all non-pandemic-related deaths. Stroke, a significant neurological disease, presents with a sudden onset followed by chronic disease [2]. It affects individuals of all age groups, with a significant impact on the elderly population [3].

According to the World Health Organization (WHO), stroke was the third most common cause of mortality globally in 2021, contributing to approximately 10% of all deaths. In lower-middle-income countries, such as India, it remains the third leading cause of death, posing a significant burden on developing economies [4]. India reported 1.44-1.66 million new stroke cases annually [5]. Factors associated with post-stroke mortality are advanced age, gender, type of stroke, severity of stroke, post-stroke infections, and comorbidities like diabetes and heart disease [6-8]. Ageing involves a gradual and irreversible decline in the physiological functions of all organ systems. Adhering to complex medication regimens is especially challenging for the elderly due to factors such as forgetfulness, lack of familial support, financial constraints, and potential adverse drug reactions or interactions [9]. Further existence of multiple risks like comorbidity, polypharmacy, lifelong medication and medication errors, makes routine monitoring mandatory in elderly post-stroke patients [10].

The WHO defines medication adherence as “the degree to which a person's behavior corresponds with the agreed recommendations from the health care provider” [11]. Adherence to the prescribed medications is the responsibility of not only the patient but also the treating physician and the healthcare system [12]. It is also influenced by various sociodemographic and medication factors. Adherence rates for chronic diseases are estimated to be around 50%, as per WHO data [12]. Lack of medication adherence heightens the risk of disability and mortality, especially in elderly stroke patients, where recurrent attacks can be life-threatening or may result in complete physical dependence [12]. Disability not only causes suffering for the patient but also contributes to significant depression and anxiety among family members [13]. Poor medication adherence could result in reduced treatment efficacy, adverse outcomes, diminished quality of life, and increased financial burdens on patients and healthcare systems. This issue poses a significant challenge, particularly for developing economies like India [9].

Numerous studies on medication adherence in geriatric populations with chronic diseases have been conducted in both developed [14,15] and developing nations [16,17]. However, only a few studies have been reported from India [9,18], and none have specifically focused on post-stroke elderly patients. Therefore, the present study was undertaken to analyze medication adherence in post-stroke elderly patients and the relationship of associated factors with medication adherence levels.

## Materials and methods

Study design and setting

This was a cross-sectional, questionnaire-based observational study conducted at the Geriatric outpatient department (OPD) of a tertiary care government hospital in Rajasthan, India.

Study population and sample size

The study included 100 post-stroke patients of both sexes, aged over 60 years, visiting the Geriatric OPD. These patients had a history of one or more episodes of clinically diagnosed stroke, either ischemic or hemorrhagic, that occurred at least six months before the study, regardless of complications. The patients had been on stroke medications for at least six months. After six months, they move beyond the acute phase, during which the urge to take medications is strongest and usually stabilizes. Some recover fully, while others live with chronic effects; therefore, a six-month cut-off was selected [19]. Patients who fulfilled the criteria mentioned above and were willing to provide written informed consent were selected for the study. Patients newly diagnosed or recently started on medications were excluded from the study.

The sample size was calculated using Cochran’s formula, based on a stroke prevalence of 6% in the Geriatric OPD, as reported in a published article [20]. The confidence interval was set at 95% with a 5% margin of error. The minimum effective sample size was calculated to be 100, including a 5% allowance for non-respondents.

Data collection method

Ethical approval was obtained from the Institutional Ethical Committee, Rabindra Nath Tagore Medical College and Hospitals, Udaipur, India (ACAD/IEC/2024/414). Patients visiting the Geriatric OPD, who met the inclusion criteria, were randomly selected for the study using a convenient sampling method. Participants were briefly informed about the study objectives in the local language, ensuring clarity and understanding. They were informed that participation was voluntary, and they could withdraw at any stage without consequences. Written informed consent was obtained from all participants. The confidentiality of participants was maintained by assigning anonymous serial numbers. Data were collected via face-to-face structured interviews during OPD visits of patients, utilizing a pre-validated questionnaire, by the principal investigator, who understood the purpose of the study and was well-versed in both the content of the questionnaire and interview techniques.

Study tools

The study questionnaire used during the interviews was prepared after a thorough literature review of related studies [9,12,16,18]. It was validated by the faculty members from the Department of Pharmacology and Medicine. The questionnaire was divided into three sections, and the interview took approximately 10 to 15 minutes to complete. The first section included nine questions and consisted of personal and demographic details, including age, gender, marital status, spouse status (alive and living with the patient, or deceased), family type, education level, occupation, and total family income per month.

The second section included twelve questions covering present and past illnesses, as well as information about medications, such as the total number of medications per day (with fixed-dose combinations considered a single medication), maximum daily drug frequency, the route of drug administration, time since commencement of stroke therapy, patient knowledge of medications, person administering the medications, patient beliefs about the safety and efficacy of medications, and concurrent use of any complementary and alternative medicines.

The third section used the Adherence to Refills and Medications Scale (ARMS) [21]. This scale uses simple language and is easy for patients with low literacy levels. It is a validated and reliable tool for assessing adherence among chronic disease patients. The ARMS is a 12-item, four-point Likert scale that evaluates self-reported adherence to refilling and taking medications. Each item had four Likert scale responses: “none,” “some,” “most,” or “all” of the time, with values ranging from one to four. One of the questions, which relates to the cost of medicine, was not included as drugs are provided free of charge at our hospital. Another question, related to ‘running out of medicines’, overlapped with another item and was therefore excluded. Thus, 10 out of 12 items were used in the study. Eight items assessed adherence to medication intake, while the remaining two items assessed adherence to refilling medications on time. Most of the items were structured so that a lower score indicated better adherence, except for a refilling item, which was reverse-coded [21].

The total possible score on the ARMS ranges from 10 to 40, where a lower score indicates better adherence and a higher score indicates poor adherence. A score of ≤ 13 was used as the cut-off point to categorize participants as “adherent (≤13)” or “non-adherent (>13).”

Statistical analysis

Qualitative data were presented as frequency and percentage, including variables such as age, gender, marital status, education, occupation, family type, family income, and various medication- and ailment-related factors. Quantitative data were presented as mean ± standard deviation (SD) and interquartile range (IQR) for variables like age and ARMS scores. The association between various factors and medication adherence was assessed using the chi-square test, and a p-value < 0.05 was considered statistically significant. Fisher’s exact test was used when the chi-square test was not applicable due to small sample sizes (expected frequency less than or equal to 5). Data were entered in Microsoft Excel 11.0 (Microsoft, Washington, DC), statistical analysis and graphical representations were performed using SPSS version 21.0 (IBM Corp., Armonk, NY).

## Results

Demographic details of participants

Out of 100 participants enrolled, 75% were male and 25% female. The mean age was 67.58 years (SD± 6.78) and 72% of participants belonged to the age group of 60-69 years (Table 1).

**Table 1:** Age and gender-wise distribution of participants (n=100).

Age group (years)	Number of males, n (%)	Number of females, n (%)	Total number of participants, n (%)
60-69	57 (79.1)	15 (20.9)	72 (72)
70-79	14 (66.6)	7 (33.3)	21 (21)
≥ 80	4 (57.1)	3 (42.9)	7 (7)
Total number of participants, n (%)	75 (75)	25 (25)	100 (100)
Mean ± SD	66.86±6.24	69.72±7.81	67.58±6.78

Ninety-eight percent of participants were married, with 86% having their spouses alive and living with them. Around 84% of participants lived in joint families, while 16% lived either in a nuclear family or alone. About 42% were illiterate, 51% had school-level education, and 7% had graduate or higher-level education. Twenty-eight percent of participants were unemployed and not involved in any income-generating outdoor activities. A statistically significant association was found between medication adherence and family type (p=0.0309), education level (p=0.0016), and the occupation of participants (p=0.0323) (Table 2).

**Table 2:** Medication adherence levels based on sociodemographic factors and their association (n=100). p-value < 0.05 considered as statistically significant. *Values presented as number (%). **Total number is not 100 as only married participants taken (n=98). ^a^Factors found to be significant with p-value <0.05. ^b^Fisher’s exact test applied as expected frequency ≤ 5. HS, Higher secondary; PG, Postgraduate.

Factors/ variables		Adherence level*		Total number of participants (n)	Chi-square value	P-value
		Adherent, n (%)	Non-adherent, n (%)			
Age (years)	60-69	24 (33.3)	48 (66.6)	72	0.0129	0.9094
	≥70	9 (32.1)	19 (67.8)	28		
Gender	Male	28 (37.3)	47 (62.6)	75	1.8242	0.1768
	Female	5^b^ (20)	20 (80)	25		
Marital status	Married	33 (33.7)	65 (66.3)	98	1	> 0.05
	Unmarried	0^b^ (0)	2^b^ (100)	2		
Family type^a^	Joint	24 (28.5)	60 (71.4)	84	4.6569	0.0309^a^
	Nuclear/alone	9 (56.2)	7 (43.7)	16		
Spouse**	Alive and living with patient	27 (32.1)	57 (67.9)	84	0.2303	0.63127
	Dead/not living with patient	6 (42.9)	8 (57.1)	14		
Education^a^	Illiterate	8 (19.1)	34 (80.9)	42	15.1481	0.0016^a^
	Primary	5^b^ (25)	15 (75)	20		
	Secondary/HS	14 (45.1)	17 (54.8)	31		
	Graduate/PG	6 (85.7)	1^b^ (14.3)	7		
Occupation^a^	Unemployed	5^b^ (17.9)	23 (82.1)	28	8.7794	0.0323^a^
	Farmer	11 (28.2)	28 (71.8)	39		
	Skilled/semi-skilled	15 (53.6)	13 (46.4)	28		
	Professional/semi-professional	2^b^ (40)	3^b^ (60)	5		
Family income (rupees/month)	< 10,000	11 (36.7)	19 (63.3)	30	0.2779	0.8702
	10,001-20,000	12 (30.7)	27 (69.3)	39		
	>20,000	10 (32.2)	21 (67.8)	31		

Adherence to refills and medications scale

**Figure 1:**
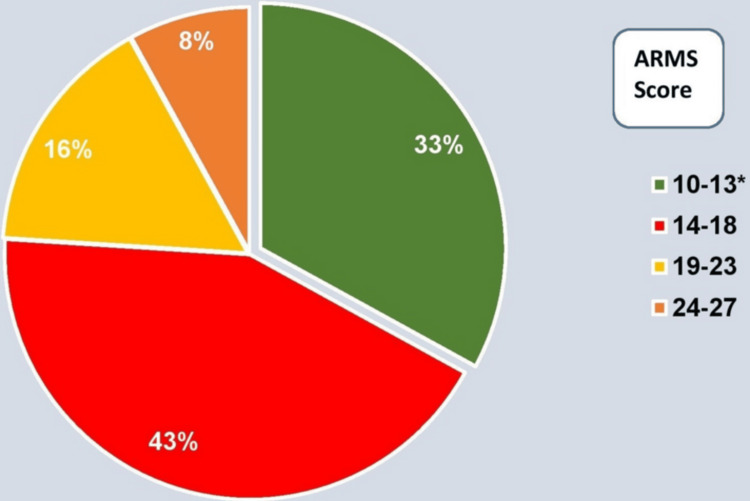
Distribution of participants based on adherence to refills and medications scale (ARMS) score categories (n=100). *, ARMS score ≤13 considered Adherent (33%). Values are represented in percentages.

ARMS scores ranged from 10 to 27, with a mean score of 15.72. Of the total participants, 33% were adherent (ARMS score ≤13) and 67% were non-adherent (ARMS score >13) (Figure 1).

Ailment and medication details

About 87% of participants had ischemic strokes, while 13% experienced hemorrhagic strokes. Around 50% of participants had a single comorbidity, while 35% had two or more comorbidities. Hypertension was the most common comorbidity, found in 74% of participants, followed by diabetes in 24%, cardiac disease in 11%, and thyroid disorders in 7%.

About 97% of participants were taking medications orally, while 3% were taking medicines through both oral and parenteral (injectable) routes. Around 19% of participants were taking up to four medicines daily, while 78% were taking five to 10 medicines daily. Polypharmacy was prevalent in 81% of participants. Also, 4% of participants reported using any type of complementary or alternative medicine alongside modern medicines.

Adherence level and medication/ailment factors

Medication adherence was 40% among participants who had sufficient knowledge about their medications, compared to 24.4% among those with limited knowledge, although the difference was not statistically significant. When participants or their spouses managed their medication, adherence was higher compared to those who were dependent on other family members, with this association being statistically significant (p=0.0020).

Around 85% of the participants believed that their medications were effective, safe and essential, whereas 15% expressed concerns that medications, although essential might be harmful. This belief was reflected in their medication adherence, as adherence was high (37.6%) among those who believed medications were beneficial and low (6.6%) among those with concerns. This finding was also statistically significant (p=0.0398) (Table 3).

**Table 3:** Medication adherence levels based on ailment or medication related factors and their association (n=100). P-value < 0.05 considered statistically significant. * Values presented as number (%). ^a^Factors found to be significant with p-value < 0.05. ^b^Fisher’s exact test applied as expected frequency ≤ 5. CAM, Complementary and alternative medicines.

Factors/variables		Adherence level*		Total number of participants (n)	Chi-square value	P-value
		Adherent, n (%)	Non-adherent, n (%)			
Type of stroke	Ischemic	29 (33.3)	58 (66.7)	87	0.0176	0.8943
	Hemorrhagic	4^b^ (30.8)	9 (69.2)	13		
Number of comorbidities	None	4^b^ (26.7)	11 (73.3)	15	1.1479	0.5632
	One	19 (38)	31 (62)	50		
	≥Two	10 (28.6)	25 (71.4)	35		
Time since start of stroke therapy	6 months-1 year	12 (32.4)	25 (67.6)	37	0.5192	0.7713
	>1 year to 3 years	9 (29)	22 (71)	31		
	> 3 years	12 (37.5)	20 (62.5)	32		
Number of medications prescribed	One to four	5^b^ (26.3)	14 (73.7)	19	0.474	0.4911
	≥ five	28 (34.6)	53 (65.4)	81		
Maximum frequency of drug per day	Once	15 (32.6)	31 (67.4)	46	1.1858	0.5527
	Twice	14 (30.5)	32 (69.5)	46		
	Thrice	4^b^ (50)	4^b^ (50)	8		
Route of drug administration	Oral	32 (32.9)	65 (67.1)	97	0.3732	0.5412
	Oral with injection	1^b^ (33.3)	2^b^ (66.7)	3		
Knowledge of drugs prescribed	Yes	22 (40)	33 (60)	55	2.7087	0.0998
	No/partial	11 (24.4)	34 (75.6)	45		
Drug administering person^a^	Self/spouse	32 (41.6)	45 (58.4)	77	9.4717	0.0020^a^
	Other family member	1^b^ (4.3)	22 (95.7)	23		
CAM intake	Yes	1^b^ (25)	3^b^ (75)	4	0.0382	0.8451
	No	32 (33.3)	64 (66.7)	96		
Patient’s belief about medications^a^	Safe, effective	32 (37.6)	53 (62.4)	85	4.2222	0.0398^a^
	Harmful but essential	1^b^ (6.67)	14 (93.4)	15		

## Discussion

Adhering to prescribed medications is vital for achieving desired therapeutic outcomes. The persistent nature of the illness, the requirement for intricate drug therapies, side effects, potential drug interactions, memory issues, and a lack of adequate family or social support can all contribute to poor adherence to long-term medication plans among elderly individuals [22]. Post-stroke elderly patients are at high risk of cognitive and motor complications, making medication adherence even more crucial for them [23]. The majority of studies from India have focused on medication adherence in elderly patients with chronic diseases [9,18,24,25]. To the best of our knowledge, this is the first Indian study focusing on elderly post-stroke patients.

Most of the patients in the study were aged between 60 and 69 years, were married, and lived in joint families. Also, 42% were illiterate, and nearly half had a school-level education. We used the ARMS score to assess medication adherence, as the ARMS scale is a validated and reliable tool for assessing adherence among chronic disease patients [21]. Only 33% of participants were found to be adherent, while 67% were non-adherent. Other studies from India and other countries have also reported low adherence levels among elderly patients with few exceptions [9,14,24,26]. A similar Indian study on post-stroke patients, but not limited to the elderly, has reported 43% adherence [27]. Punnapurath et al., in their study from South India, reported a high adherence level of 82% among geriatric chronic disease patients [25]. A recent study from China on elderly post-stroke patients reported non-adherence in 61.4% of patients [17]. Another study from Saudi Arabia, not limited to elderly patients, reported 83% non-adherence [16]. The median ARMS score was 15.72 in our study, which is 2.72 points higher than the cutoff point (ARMS score ≤13 considered adherent). A study from Kuwait on geriatric patients with chronic diseases reported a median ARMS score of 20, which indicates lower adherence than that reported by us [28].

Hypertension (74%) was the most common comorbidity, followed by diabetes mellitus (24%) and cardiac diseases (11%). Other studies on elderly patients with chronic diseases have reported similar results [9,25]. Comorbidities increase the prevalence of polypharmacy and a complex drug regimen. Polypharmacy (81%) was prevalent in our study as well.

Age of the patients was not significantly associated with adherence in our study. Few authors have reported that adherence decreases with an increase in age [9,22,24]. Males (37.3%) were more adherent than females (20%), though the difference was not statistically significant. Similar results have also been reported by other authors [9,26].

A statistically significant association was found between medication adherence and family type (p=0.0309). In our study, patients living in nuclear families were more adherent compared to those living in joint families. Joint families have multiple members, and if the responsibility of giving medicines to the elderly is not pre-decided, compliance with prescribed medicines becomes difficult. Shruthi et al. have also reported that medication adherence was better in patients living with their spouse as compared to those living with extended family [9].

Patients with a higher education level were found to have better adherence (p=0.0016), which agrees with other authors [9,14,16,17,27]. Educated patients can better understand the nature of the disease, the complexity of the drug regimen, as well as the risks associated with non-adherence. Similarly, occupation of participants also affected adherence significantly (p=0.0323). Patients with skilled/semi-skilled professions were better adherent as compared to farmers and unemployed patients. This is also indirectly related to education, as skilled people are usually more educated than farmers.

Medication adherence was significantly higher when drug administration was the responsibility of the patient or spouse, as compared to other family members (p=0.002). This finding coincides with the finding that patients living in nuclear families had better adherence than those living in joint families. When a patient or their spouse takes the responsibility of drug administration, they tend to be more adherent. This is largely due to greater personal interest, emotional involvement, and a better understanding of the treatment plan. They are more motivated, maintain consistent routines, and communicate more effectively. In contrast, other family members may lack the same level of engagement. Similarly, patients who have a positive belief toward the recommended regimen had better adherence as compared to those who consider them harmful (p=0.0398). These findings suggest that educated patients, who better understand the disease and the prescribed drugs, have a positive attitude towards medicines and take self-responsibility for drug administration, are more likely to be adherent. These facts also indicate that uneducated, unemployed patients and those living in extended families need extra emphasis and counselling to comply with proper and regular intake of drugs.

Our study findings suggest that the overall medication adherence is low in geriatric post-stroke survivors. They are at high risk of complications from such diseases due to poor adherence, comorbidities, and deteriorating physical and mental status. Consistent medication adherence is highly crucial for the successful management of neurological conditions like post-stroke, as well as for the prevention of complications and risk management.

Frequent medication reviews by physicians, better collaboration between patients, patient’s family members, and healthcare providers, and the use of dosing aids are some of the strategies that could improve medication adherence in the elderly [29]. Dosing aids include medication calendars, drug cards, charts, pill boxes, and specialized boxes indicating the dose time. The selection of appropriate dosing assistance should be based on the individual patient’s requirements [11]. Patients and family members should be sensitized regarding the necessity of timely intake of drugs and the risks of medication non-adherence. If needed, a collaborative approach involving pharmacists and other healthcare professionals should be adopted at hospitals to educate patients and ensure better adherence.

Limitations

As this study relied on self-reported responses, the potential for recall or self-reporting bias cannot be excluded. The analysis in the present study was conducted using the total ARMS score only, and not based on two different subdomains- adherence and refilling. Additionally, the relatively small sample size and the recruitment of participants from a single center limit the generalizability of the findings. Future research with larger, more diverse samples is warranted to enhance understanding of the associated factors and improve the applicability of the results. Moreover, the impact of financial factors on medication adherence could not be assessed, as the study was conducted in a government hospital setting where medications are provided free of charge.

Strength

There is a dearth of literature on medication adherence among post-stroke geriatric patients. We were not able to find any Indian studies, to the best of our knowledge. Thus, ours is the first study from India in which we have focused on medication compliance in post-stroke elderly patients and have also analyzed associated sociodemographic, ailment, and medication-related factors.

## Conclusions

Medication adherence among post-stroke elderly patients in India is significantly low. Lack of education, unemployment, and reduced familial support, particularly in joint families, adversely affect medication compliance. Patient's dependence on family members other than their spouse for drug intake and misconceptions about medications also affect adherence towards drug therapy.

Physicians must remain vigilant regarding poor adherence in elderly patients. Effective strategies, such as regular medication reviews, patient education, and counselling for both patients and their families about the importance of consistent drug intake and the risks associated with non-adherence, should be prioritized. Special emphasis should be placed on addressing patients’ fears and dispelling any false beliefs about medications. By fostering an open dialogue, healthcare providers can improve medication adherence in the long term.

## Appendices

Data collection proforma

**Table 4:** Questionnaire regarding demographic details of the participants. HS, Higher secondary; PG, Postgraduate.

Sr. No.	Demographic details	Responses					
1	Serial number						
2	Age (years)						
3	Gender	Male			Female		
4	Marital status	Married			Unmarried		
	If married, spouse	Alive or living with the patient			Dead		
5	Family type	Joint	Nuclear		Alone		Old age home
6	Education	Illiterate	Primary		Secondary/HS		Graduate/PG
7	Occupation	Unemployed/housewife	Farmer		Skilled/semi-skilled		Professionals
8	Family income per month (Indian rupees)	<10.000		10,001-20,000		>20,000	

**Table 5:** Questionnaire regarding medication and ailment details of the participants. CAM, Complementary and alternative medicines.

Sr. no.	Medication & ailment factors	Responses								
1	Type of stroke	Ischemic				Hemorrhagic				
2	Associated comorbid conditions									
3	Time since commencement of therapy	6 months-1 year		>1 year-3 years				>3 years		
4	Total number of medications prescribed for the above-mentioned ailments?	1-4		5-10				>10		
5	Maximum number of frequency (per day) of any of above-mentioned medications?	Once a day		Twice a day				Thrice a day		
6	Route of administration of the above medications?	Oral	Oral with injection			Oral with inhalational				Others
7	Whether the patient has right knowledge of the drugs prescribed to him/her?	Yes		No				Partial		
8	Person administering medications to the patient?	Self		Spouse				Other family member/caregiver		
9	Do you take any CAM, e.g., ayurveda/homeopathy/naturopathy medicine?	Yes					No			
10	What are your beliefs about modern medications you are taking?	Effective, essential and safe			Effective, essential but might be harmful				Ineffective and harmful	

Adherence to refills and medications scale (ARMS)

**Table 6:** Adherence to refills and medications scale questions with possible responses.

Questions	None	Some	Most	All
1. How often do you forget to take your medicine?	1	2	3	4
2. How often do you decide not to take your medicine?	1	2	3	4
3. How often do you forget to get prescriptions filled?	1	2	3	4
4. How often do you skip a dose of your medicine before you go to the doctor?	1	2	3	4
5. How often do you miss taking your medicine when you feel better?	1	2	3	4
6. How often do you miss taking your medicine when you feel sick?	1	2	3	4
7. How often do you miss taking your medicine when you are careless?	1	2	3	4
8. How often do you change the dose of your medicines to suit your needs (taking more or less pills than you are supposed to)?	1	2	3	4
9. How often do you forget to take your medicine when you are supposed to take it more than once a day?	1	2	3	4
10. How often do you plan ahead and refill your medicines before they run out?	1	2	3	4
